# Supplementary material for: Diet, Physical Activity, and Disinhibition in Middle-Aged and Older Adults: A UK Biobank Study
Source: Nutrients. 2021 May 11;13(5):1607. doi: 10.3390/nu13051607 (PMC8151887; doi:10.3390/nu13051607)
Supplement: Supplementary file 1 [file nutrients-13-01607-s001.zip › nutrients-1134259-supplementary.pdf]

Supplemental information

belonging to publication:

LJS Schweren, D van Rooij, H Shi, A Arias-Vasquez, L Li, H Larsson, L Grimstvedt Kvalvik, J Haavik, J Buitelaar & CA Hartman (2020). Diet, physical activity and disinhibition in middle-aged and older adults: a UK Biobank study.

Section 1: PCA to derive dietary components (DC1-4) and dietary groups (DC1-5)

Table S1. UK Biobank items related to diet. Note that items related to alcohol intake are not included, since heavy drinking contributed to our outcome of interest.

**Type**	**UKB ID**	**Item**	**Unit**
Continuous	1289	Cooked vegetables	Tablespoons / day
	1299	Raw vegetables	Tablespoons / day
	1309	Fresh fruit	Pieces / day
	1319	Dried fruit	Pieces / day
	1438	Bread	Slices / week
	1458	Cereal	Bowls / week
	1488	Tea	Cups / day
	1498	Coffee	Cups / day
	1528	Water	Glasses / day
Frequency	1329	Oily fish	Times / week [a]
	1339	Non-oily fish	Times / week [a]
	1349	Processed meat	Times / week [a]
	1359	Poultry	Times / week [a]
	1369	Beef	Times / week [a]
	1379	Lamb	Times / week [a]
	1389	Pork	Times / week [a]
	1408	Cheese	Times / week [a]
	1478	Added salt	Never or rarely / sometimes / usually / always [b]
Type [c]	1418	Milk [d]	Full-cream / semi-skimmed / skimmed / soy / other non-dairy / never
	1428, 2654	Spreads	Butter / block margarine / tub margarine / benecol / olive-oil based / sunflower-based / low-fat / other / never
	1468	Cereal	Bran / biscuit / oat / muesli / refined sugar-sweetened / never
	1508	Coffee	Decaffeinated / instant / ground / other / never
	1448	Bread	Brown / white / wholegrain / other / never
Elimination [e]	6144	I never eat (tick box)	Eggs / dairy / wheat / sugar

a. converted to days/month for quantitative processing. b. converted to 1/0.33/0.67/1 for quantitative processing. c. binary contrasts were created for each food type variable, such that each response category contrasted with all other categories and that the never category contrasted with all other. d. an additional ordinal variable was created for fat content of milk ('no milk', 'skimmed milk', 'semi-skimmed milk', 'full-cream milk'). e. each restriction was contrasted against having no restrictions and against having no restrictions or other restrictions.

Table S2. Restructured items included in the PCA, and their factor loadings in the final model with four principal components (DC1-4)

	% endorsed	DC1	DC2	DC3	DC4
Cooked vegetables	NA	0.38	0.11	0.16	-0.05
Raw vegetables / salad	NA	0.45	0.09	0.07	-0.04
Fresh fruit	NA	0.45	0.04	-0.04	-0.09
Dried fruit	NA	0.48	0.00	-0.05	0.07
Bread	NA	-0.12	-0.26	-0.03	0.05
Cereal	NA	0.31	-0.20	-0.09	0.07
Tea	NA	0.03	-0.05	0.01	0.04
Coffee	NA	0.00	-0.17	0.19	-0.09
Water	NA	0.34	0.15	0.02	-0.01
Oily fish	NA	0.47	0.07	0.27	-0.09
Non-oily fish	NA	0.33	0.02	0.33	-0.11
Processed meat	NA	-0.24	-0.07	0.48	0.05
Poultry	NA	-0.07	0.01	0.40	-0.11
Beef	NA	-0.05	0.00	0.73	0.02
Lamb	NA	0.09	0.04	0.75	0.05
Pork	NA	0.01	0.00	0.73	0.01
Cheese	NA	0.02	-0.05	0.01	0.25
Added salt	NA	-0.09	0.04	0.31	0.12
Fat content of milk	NA	-0.06	-0.01	0.05	0.83
Full cream milk vs. any other milk	6.0	-0.12	0.08	0.03	0.31
Semi-skimmed milk vs. any other milk	66.2	0.01	-0.11	0.06	0.55
Skimmed milk vs. any other milk	21.9	0.00	-0.11	0.01	-0.84
Soy milk vs. any other milk	4.7	0.11	0.28	-0.18	0.07
Other non-dairy milk vs. any other milk	1.2	0.00	0.20	-0.03	0.02
Milk vs. 'I never drink milk'	96.2	0.08	-0.24	-0.02	0.26
Spread vs. 'I never use spreads'	88.3	-0.16	-0.17	0.02	0.18
Butter vs. any other spread	41.8	0.05	0.09	0.15	0.29
Block margarine vs. any other spread	<0.1	-0.02	0.02	0.02	0.02
Tub margarine vs. any other spread	5.1	-0.17	0.01	-0.02	-0.01
Benecol vs. any other spread	10.1	0.06	-0.01	-0.01	-0.16
Olive-oil based spread vs. any other spread	16.2	0.11	-0.06	-0.05	-0.05
Sunflower-based spread vs. any other spread	19.3	-0.07	-0.09	-0.06	-0.14
Low-fat spread vs. any other spread	5.0	-0.05	-0.03	-0.03	-0.12
Other spread vs. any other spread	2.4	-0.04	0.15	-0.07	0.04
White bread vs. any other bread	20.6	-0.55	0.09	0.05	0.01
Brown bread vs. any other bread	10.1	-0.13	0.03	0.02	0.01
Wholegrain bread vs. any other bread	64.8	0.58	-0.19	-0.06	-0.02
Other bread vs. any other bread	4.5	-0.07	0.22	0.01	0.02
Bread vs. 'I never eat bread'	96.4	-0.04	-0.38	-0.02	0.05
Bran vs. any other cereal	16.8	0.02	-0.08	0.01	-0.09
Biscuit vs. any other cereal	15.4	-0.12	-0.07	0.01	-0.03
Oat vs. any other cereal	26.2	0.10	0.07	0.03	-0.08
Muesli vs. any other cereal	25.5	0.26	-0.02	-0.06	0.17
Refined sugar-sweetened cereal products	16.0	-0.33	0.09	0.02	0.02
Cereal vs. 'I never eat cereal'	84.2	0.28	-0.23	-0.09	0.10
Decaffeinated coffee vs. any other coffee	18.5	0.03	-0.01	-0.03	-0.12
Instant coffee vs. any other coffee	51.2	-0.31	-0.08	0.02	-0.09
Ground coffee vs. any other coffee	28.9	0.32	0.10	0.00	0.20
Other coffee vs. any other coffee	1.3	-0.02	0.02	-0.03	-0.01
Coffee vs. 'I never drink coffee'	80.6	0.10	-0.20	0.14	-0.04
'I never eat eggs' vs. no restrictions	2.5	-0.07	0.50	-0.06	0.04
'I never eat eggs' vs. no eggs restrictions	10.1	-0.07	0.24	-0.05	0.05
'I never eat dairy' vs. no restrictions	2.3	-0.03	0.64	-0.03	0.04

‘I never eat dairy’ vs. no dairy restrictions	9.4	-0.04	0.34	-0.02	0.06
‘I never eat wheat’ vs. no restrictions	2.6	-0.06	0.64	0.07	0.05
‘I never eat wheat’ vs. no wheat restrictions	10.6	-0.06	0.34	0.05	0.07
‘I never eat sugar’ vs. no restrictions [a]	16.5	0.08	-0.31	0.01	-0.07
I never eat eggs, sugar, wheat or dairy’ vs. no restrictions	19.9	0.06	0.51	0.03	-0.09

a. Contrasts ‘I never eat sugar vs. no restrictions’ and ‘I never eat sugar vs no sugar restrictions’ were collinear (r>0.85), hence the latter was removed.

Table S3. Correlations between DC1-4

	DC1	DC2	DC3	DC4
DC1: Prudent diet	1.000			
DC2: Wheat/dairy/egg elimination	0.112	1.000		
DC3: Meat	-0.121	-0.097	1.000	
DC4: Full-cream dairy	-0.107	-0.124	0.045	1.000

Section 2: Latent profile analysis to derive multivariate dietary groups (DC1-5)

Figure S1. Light's generalisation of Cohen's kappa's across one hundred permutations for models containing two to sixteen principal components. Kappa illustrates reproducibility of the model among permuted datasets and should ideally by 0.8 or higher.

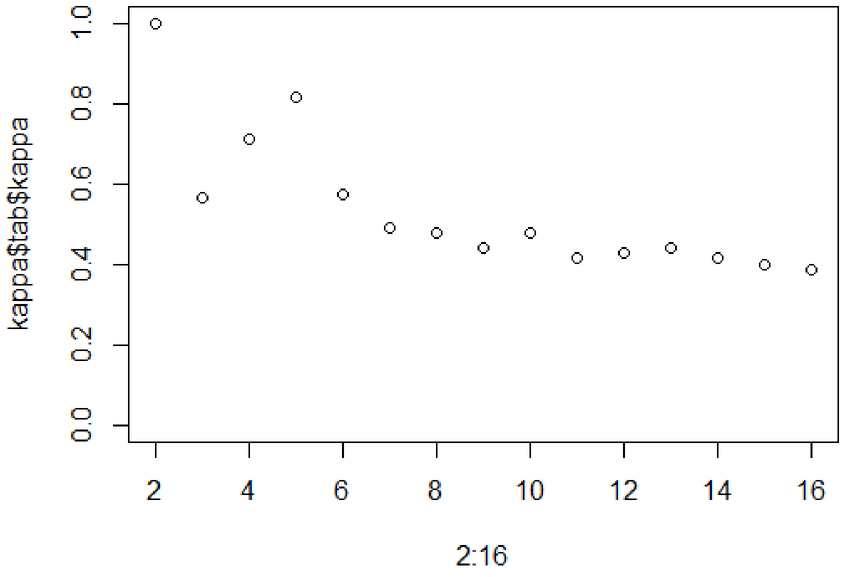

Figure S2. Increase in BIC upon adding latent participant clusters. Note that stabilisation starts after adding the fourth cluster, and is completed after adding the sixteenth cluster. VVV indicates a model with no *a priori* constraints.

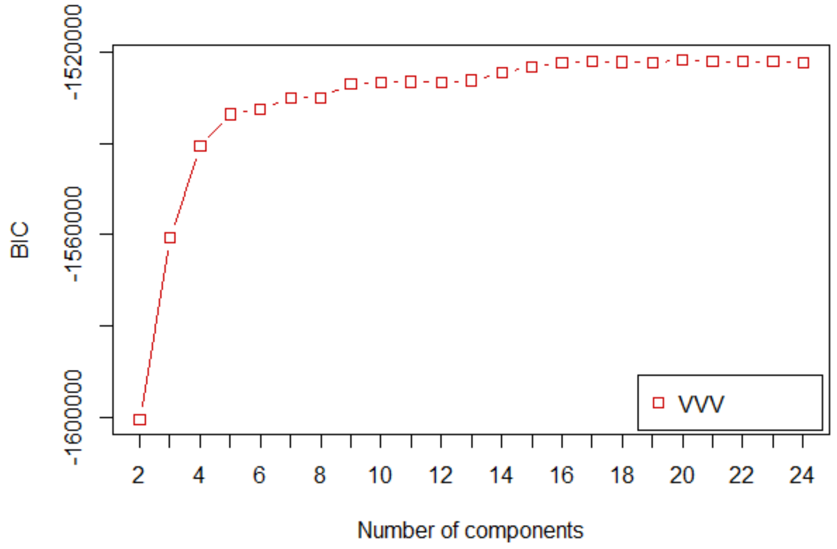

Section 3: PCA to derive behavioural disinhibition scores

First, all datafields related to disinhibition, impulsivity, compulsivity and/or emotional instability were selected by two independent authors (Supplementary tables ST3.1-3.2). Non-binary items were binarized. Next, to ascertain a balanced representation of disinhibited behaviour types, cases were defined for nine groups of disinhibited behaviours (i.e. externalising, obsessive-compulsive, addiction, self-harm, personality, mania, risk-taking, cannabis use, smoking). Case-ness was defined such that it captured relatively extreme behaviours, aiming at a 5-10% endorsement rate. For instance, participants were considered a case for addiction when they either self-reported being or having been addicted, or had at any time received a diagnosis of substance dependence syndrome in hospital, resulting in 6.1% of the sample being a case.

Next, we performed a PCA based on tetrachoric correlations between behavioural groups (ST3.3). The theoretical framework underlying our approach is that impulsive, compulsive and emotionally unstable behaviours reflect a unitary disinhibited phenotype and share a common origin. Therefore, the single-component model was preferred a priori, and more complex models were only considered if the simpler model presented with statistical or interpretational shortcomings. All behaviours loaded positively on the first principal component, with factor loadings ranging from 0.335 to 0.708 (Table 2). Models with two and three principal components are shown in ST3.4. The two-PC model differentiates between behaviours related to substance abuse (PC2) and other disinhibited behaviours (PC1), but contains double loadings for self-harm and addiction. The three-PC model adds a component reflecting unstable personality/mania, but also has double loadings (addiction, personality) and triple loadings (self-harm). Hence, the single-component model was adopted. For each subject, a factor score was extracted with higher scores indicating a higher tendency for disinhibition. Finally, after trimming at 97.5% to replace extreme values by the cut-off value (i.e. 3.4), disinhibition scores ranged from -0.78 to 3.40 (M=-0.14, SD=0.93).

Table S4. Selected UK Biobank items related to disinhibition, impulsivity, compulsivity and/or emotional instability and their binarised response categories.

Data-field	Source	Description	Response categories (original)	Response categories (binary)
1239	IAV	Do you smoke tobacco now?	- No<br>- Yes, on all/most days<br>- Only occasionally	- No / only occasionally<br>- Yes, on all/most days
1920	IAV	Does your mood often go up and down?	- No<br>- Yes	- No<br>- Yes
1940	IAV	Are you an irritable person?	- No<br>- Yes	- No<br>- Yes
1960	IAV	Do you often feel fed-up?	- No<br>- Yes	- No<br>- Yes
20544	MHQ	Have you been diagnosed with one or more of the following mental health problems by a professional, even if you don't have it currently? (tick box)	- OCD<br>- Mania<br>- ADHD	- No<br>- Yes<br>(per diagnosis)
20548	MHQ	Try to remember a period when you were in a "high" or "irritable" state (tick box) (conditional to: ever had period of extreme irritability/excitability)	- More restless than usual<br>- Thoughts were racing<br>- Easily distracted<br>- More active than usual	- No<br>- Yes, ever had period of mania/irritability with at least one of these symptoms
20401	MHQ	Have you been addicted to or dependent on one or more things, including substances (not cigarettes/coffee) or behaviours (such as gambling)?	- No<br>- Yes	- No<br>- Yes
20416	MHQ	How often do you have six or more drinks on one occasion?	- Never<br>- Less than monthly	- Never / less than weekly

		(conditional to: frequency of drinking alcohol)	- Monthly<br>- Weekly<br>- (Almost) daily	- Weekly / (almost) daily
20453	MHQ	Have you taken cannabis (marijuana, grass etc.), even if it was a long time ago?	- No<br>- Yes, 1-2 times<br>- Yes, 3-10 times<br>- Yes, 11-100 times<br>- Yes, more than 100 times	- No / less than 11 times<br>- Yes, 11 times / more
20480	MHQ	Have you deliberately harmed yourself, whether or not you meant to end your life?	- No<br>- Yes	- No<br>- Yes
41202 and 41204	LHR	Main or secondary ICD-10 diagnoses from hospital inpatient records	See supplementary table ST3.2	- No<br>- Yes<br>(per diagnostic group)

Abbreviations: IAV= initial assessment visit, MHQ= mental health questionnaire, LHR= linked health records, ICD-10= international classification of diagnoses, 10th edition.

Table S5. ICD-10 diagnoses related to impulsivity, compulsivity and/or emotional instability per diagnostic group.

Addiction	F102	Dependence syndrome of alcohol
	F112	Dependence syndrome of opioids
	F122	Dependence syndrome of cannabinoids
	F132	Dependence syndrome of sedatives or hypnotics
	F142	Dependence syndrome of cocaine
	F152	Dependence syndrome of other stimulants
	F162	Dependence syndrome of hallucinogens
	F192	Dependence syndrome of multiple drug use and use of other psychoactive substances
Externalising	F900	Disturbance of activity and attention
	F901	Hyperkinetic conduct disorder
	F908	Other hyperkinetic disorders
	F909	Hyperkinetic disorder unspecified
	F910	Conduct disorder confined to the family context
	F911	Unsocialised conduct disorder
	F912	Socialised conduct disorder
	F913	Oppositional defiant disorder
	F918	Other conduct disorders
	F919	Conduct disorder unspecified
	F920	Depressive conduct disorder
	F928	Other mixed disorders of conduct and emotions
	F929	Mixed disorder of conduct and emotions unspecified
Mania	F300	Hypomania
	F301	Mania without psychotic symptoms
	F302	Mania with psychotic symptoms
	F308	Other manic episodes
	F309	Manic episode unspecified
	F310	Bipolar affective disorder current episode hypomanic
	F311	Bipolar affective disorder current episode manic without psychotic symptoms
	F312	Bipolar affective disorder current episode manic with psychotic symptoms
	F313	Bipolar affective disorder current episode mild or moderate depression
	F314	Bipolar affective disorder current episode severe depression w/o psychotic symptoms
	F315	Bipolar affective disorder current episode severe depression with psychotic symptoms
	F316	Bipolar affective disorder current episode mixed
	F317	Bipolar affective disorder currently in remission
	F318	Other bipolar affective disorders
	F319	Bipolar affective disorder unspecified
OCD	F420	Predominantly obsessional thoughts or ruminations
	F421	Predominantly compulsive acts [obsessional rituals]
	F422	Mixed obsessional thoughts and acts
	F428	Other obsessive-compulsive disorders
	F429	Obsessive-compulsive disorder unspecified
	F633	Trichotillomania
	F950	Transient tic disorder
	F951	Chronic motor or vocal tic disorder

	F952	Combined vocal and multiple motor tic disorder [de la Tourette]
	F958	Other tic disorders
	F959	Tic disorder unspecified
	F605	Anankastic personality disorder
Personality	F603	Emotionally unstable personality disorder
Risk-taking	F100	Acute intoxication with alcohol
	F101	Harmful use of acohol
	F110	Acute intoxication of opioids
	F111	Harmful use of opioids
	F120	Acute intoxication cannabinoids
	F121	Harmful use cannabinoids
	F130	Acute intoxication of sedatives or hypnotics
	F131	Harmful use of sedatives or hypnotics
	F140	Acute intoxication of cocaine
	F141	Harmful use of cocaine
	F150	Acute intoxicationof other stimulants
	F151	Harmful use of other stimulants
	F160	Acute intoxication of hallucinogens
	F161	Harmful use of hallucinogens
	F190	Acute intoxication due to multiple drug use and use of other psychoactive substances
	F191	Harmful use due to multiple drug use and use of other psychoactive substances
	F630	Pathological gambling
	F631	Pathological fire-setting [pyromania]
	F632	Pathological stealing [kleptomania]
	F638	Other habit and impulse disorders
	F639	Habit and impulse disorder unspecified

Table S6: Number of cases per disinhibited behaviour group, their sensitivity and factor loadings.

Group	N (%)	Loading	Definition
Addiction	9,534 (6.1)	0.708	a) participant self-reports past or current addiction – OR –<br>b) hospital diagnosis of substance dependence syndrome
Self-harm	6,871 (4.4)	0.637	a) participant self-reports past or current self-harm
Mania	25,763 (16.4)	0.613	a) participant self-reports diagnosis of mania by professional – OR –<br>b) hospital diagnosis of mania/bipolar disorder – OR –<br>c) participant has experienced period with at least one symptom of mania
OCD	996 (0.6)	0.591	a) participant self-reports diagnosis of OCD by professional – OR –<br>b) hospital diagnosis of OCD/tic disorder/trichotillomania/anankastic PD
Externalising	134 (0.1)	0.589	a) participant self-reports diagnosis of ADHD by professional – OR –<br>b) hospital diagnosis of any externalising disorder
Cannabis use	11,205 (7.1)	0.552	a) participant self-reports having used cannabis >10 times in life
Personality	23,892 (15.2)	0.536	a_1) participant self-reports moods going up and down – AND –<br>a_2) participant self-reports being easily fed-up – AND –<br>a_3) participant self-reports being irritable – OR –<br>b) hospital diagnosis of emotionally unstable PD
Smoking	7,615 (4.8)	0.475	a) participant self-reports current daily smoking
Risk-taking	23,994 (15.3)	0.335	a) participant self-reports weekly binge-drinking – OR –<br>b) hospital diagnosis of acute intoxication with alcohol/drugs

Abbreviations: ADHD = attention-deficit/hyperactivity disorder; OCD = obsessive-compulsive disorder; PD = personality disorder

Table S7. Tetrachoric correlations between the nine disinhibited behaviour groups.

	OCD	Mania	Externalising	Personality	Risk-taking	Smoking	Addiction	Self-harm
Mania	0.285							
Externalising	0.527	0.298						
Personality	0.276	0.413	0.214					
Risk-taking	0.000	0.090	0.048	0.098				
Smoking	0.096	0.156	0.065	0.119	0.196			
Addiction	0.278	0.295	0.287	0.221	0.298	0.357		
Self-harm	0.318	0.322	0.239	0.265	0.075	0.217	0.366	
Cannabis	0.090	0.156	0.205	0.118	0.273	0.354	0.392	0.288

Table S8. PCA results extracting one, two and three principal components. The two-PC model differentiates between behaviours related to substance abuse (PC2) and other disinhibited behaviours (PC1), but contains double loadings for self-harm and addiction. The three-PC model adds a component reflecting unstable personality/mania, but also has double loadings (addiction, personality) and triple loadings (self-harm).

	Model: 1 PC	Model: 2 PCs		Model: 3 PCs		
	PC1	PC1	PC2	PC1	PC2	PC3
OCD	0.591	0.816	-0.153	0.847		
Mania	0.613	0.634				0.788
Externalising	0.589	0.739		0.883		-0.113
Personality	0.536	0.692		-0.108	-0.103	0.925
Risk-taking	0.335	-0.223	0.686	-0.287	0.658	
Smoking	0.475		0.715	-0.107	0.712	
Addiction	0.708	0.259	0.627	0.218	0.650	
Self-harm	0.637	0.482	0.283	0.272	0.278	0.295
Cannabis	0.552		0.749		0.788	-0.167

Section 4: Outcomes when including age-by-predictor interaction terms

None of the associations reported in the main paper changed upon adding age-interaction effects (ST4.1). Moreover, most age-interaction effects were non-significant (ST4.2). In women, age-interactions with the unhealthy-vs-moderate-and-prudent, restricted-vs-moderate-and-prudent and low-fat dairy-vs-moderate-and-prudent group contrasts reached significance. In men, the age-by-DC2 interaction reached significance. For these interactions, we calculated the simple slopes of the dietary predictor for ten age-deciles (ST4.3). Note that a) the direction of effect remains the same across the age range, and b) the effect remains significant across the age range except at very late age. We conclude that some associations between diet and disinhibition may be stronger at younger age, especially in women, but that overall associations between diet and behavioural disinhibition are not moderated by age.

Table S9. Associations as reported in the main paper, after including an age-by-predictor interaction term

		**Men**			**Women**		
		**β**	**SE**	**P-value**	**β**	**SE**	**P-value**
Single-predictor	DC1	-0.036	0.004	<0.0001	-0.043	0.003	<0.0001
	DC2	0.031	0.004	<0.0001	0.038	0.003	<0.0001
	DC3	0.041	0.004	<0.0001	-0.016	0.003	<0.0001
	DC4	0.023	0.004	<0.0001	0.010	0.003	0.0036
	Unhealthy-vs-moderate-and-prudent	0.086	0.010	<0.0001	0.096	0.009	<0.0001
	Restricted-vs-moderate-and-prudent	0.125	0.016	<0.0001	0.158	0.013	<0.0001
	Avoid meat-vs-moderate-and-prudent	0.075	0.011	<0.0001	0.153	0.011	<0.0001
	Low-fat dairy-vs-moderate-and-prudent	0.041	0.011	0.0002	0.054	0.009	<0.0001
	MVPA	-0.007	0.004	0.0523	-0.009	0.003	0.0053
Multiple predictor	DC1	-0.031	0.004	<0.0001	-0.049	0.003	<0.0001
	DC2	0.040	0.004	<0.0001	0.044	0.003	<0.0001
	DC3	0.039	0.004	<0.0001	-0.018	0.003	<0.0001
	DC4	0.022	0.004	<0.0001	0.010	0.003	0.0017
	Unhealthy-vs-moderate-and-prudent	0.085	0.010	<0.0001	0.094	0.009	<0.0001
	Restricted-vs-moderate-and-prudent	0.125	0.016	<0.0001	0.157	0.013	<0.0001
	Avoid meat-vs-moderate-and-prudent	0.075	0.011	<0.0001	0.153	0.011	<0.0001
	Low-fat dairy-vs-moderate-and-prudent	0.041	0.011	0.0002	0.053	0.009	<0.0001
	MVPA	-0.004	0.004	0.2522	-0.003	0.003	0.3733

* β>0.02 & p<0.0028.

Table S10. Age-by-predictor interaction terms, added to the models as described in the main paper.

		Men			Women		
		**β**	**SE**	**P-value**	**β**	**SE**	**P-value**
Single-predictor	DC1-by-age	-0.002	0.004	0.5530	0.011	0.003	0.0007
	DC2-by-age	-0.019	0.004	<0.0001	-0.014	0.003	<0.0001
	DC3-by-age	<0.001	0.004	0.9122	0.003	0.003	0.3356
	DC4-by-age	-0.004	0.004	0.2709	0.004	0.003	0.2653
	Unhealthy-vs-moderate-and-prudent-by-age	-0.014	0.010	0.1521	-0.031	0.009	0.0003*
	Restricted-vs-moderate-and-prudent-by-age	-0.044	0.016	0.0045	-0.065	0.013	<0.0001*
	Avoid meat-vs-moderate-and-prudent-by-age	-0.032	0.011	0.0061	-0.027	0.011	0.0135
	Low-fat dairy-vs-moderate-and-prudent-by-age	-0.022	0.011	0.0466	-0.031	0.009	0.0005*
	MVPA-by-age	-0.005	0.004	0.1422	0.001	0.003	0.6528
Multiple predictor	DC1-by-age	0.004	0.004	0.2564	0.013	0.003	0.0001
	DC2-by-age	-0.021	0.004	<0.0001*	-0.015	0.003	<0.0001
	DC3-by-age	-0.002	0.004	0.6387	0.002	0.003	0.4604
	DC4-by-age	-0.007	0.004	0.0486	0.003	0.003	0.3806
	Unhealthy-vs-moderate-and-prudent-by-age	-0.014	0.010	0.1364	-0.030	0.009	0.0004*
	Restricted-vs-moderate-and-prudent-by-age	-0.045	0.016	0.0044	-0.065	0.013	<0.0001*
	Avoid meat-vs-moderate-and-prudent-by-age	-0.030	0.011	0.0061	-0.027	0.011	0.0130
	Low-fat dairy-vs-moderate-and-prudent-by-age	-0.023	0.011	0.0451	-0.031	0.009	0.0006*
	MVPA-by-age	-0.007	0.004	0.0821	-0.001	0.003	0.8340

* $\beta>0.02$ & $p<0.0028$.

Table S11. Simple slopes at ten age-deciles for the association between disinhibition and the dietary predictor, in those instances where the age-by-dietary predictor interaction term reached significance.

			Single-predictor model			Multiple-predictor model		
		Age (SD)	β	SE	P-value	β	SE	P-value
Women	Unhealthy-vs-moderate-and-prudent	-2.0	0.151	0.019	<0.0001	0.149	0.019	<0.0001
		-1.6	0.138	0.016	<0.0001	0.136	0.016	<0.0001
		-1.1	0.124	0.013	<0.0001	0.123	0.013	<0.0001
		-0.7	0.111	0.010	<0.0001	0.110	0.010	<0.0001
		-0.3	0.098	0.009	<0.0001	0.097	0.009	<0.0001
		0.2	0.085	0.009	<0.0001	0.084	0.009	<0.0001
		0.6	0.072	0.010	<0.0001	0.071	0.010	<0.0001
		1.0	0.059	0.012	<0.0001	0.058	0.012	<0.0001
		1.5	0.046	0.015	0.0026	0.045	0.015	0.0031
		1.9	0.032	0.018	0.0754	0.032	0.018	0.0823
	Restricted-vs-moderate-and-prudent	-2.0	0.288	0.030	<0.0001	0.288	0.030	<0.0001
		-1.6	0.260	0.025	<0.0001	0.260	0.025	<0.0001
		-1.1	0.232	0.020	<0.0001	0.232	0.020	<0.0001
		-0.7	0.204	0.016	<0.0001	0.204	0.016	<0.0001
		-0.3	0.176	0.014	<0.0001	0.175	0.014	<0.0001
		0.2	0.147	0.013	<0.0001	0.147	0.013	<0.0001
		0.6	0.119	0.015	<0.0001	0.119	0.015	<0.0001
		1.0	0.091	0.018	<0.0001	0.091	0.018	<0.0001
		1.5	0.063	0.022	0.0049	0.063	0.022	0.0051
		1.9	0.035	0.027	0.1996	0.035	0.027	0.2026
	Low-fat dairy-vs-moderate-and-prudent	-2.0	0.111	0.019	<0.0001	0.110	0.019	<0.0001
		-1.6	0.098	0.016	<0.0001	0.097	0.016	<0.0001
		-1.1	0.085	0.013	<0.0001	0.085	0.013	<0.0001
		-0.7	0.072	0.011	<0.0001	0.072	0.011	<0.0001
		-0.3	0.059	0.009	<0.0001	0.059	0.009	<0.0001
		0.2	0.047	0.009	<0.0001	0.046	0.009	<0.0001
		0.6	0.034	0.010	0.0006	0.033	0.010	0.0007
		1.0	0.021	0.012	0.0830	0.021	0.012	0.0898
		1.5	0.008	0.015	0.5816	0.008	0.015	0.6027
		1.9	-0.005	0.018	0.8032	-0.005	0.018	0.7833
Men	DC2	-2.4				0.090	0.010	<0.0001
		-1.9				0.080	0.008	<0.0001
		-1.4				0.070	0.007	<0.0001
		-0.9				0.059	0.005	<0.0001
		-0.4				0.049	0.004	<0.0001
		0.0				0.039	0.004	<0.0001
		0.5				0.029	0.004	0.0006
		1.0				0.019	0.005	0.0003
		1.5				0.009	0.007	0.1965
		2.0				-0.002	0.008	0.8416
